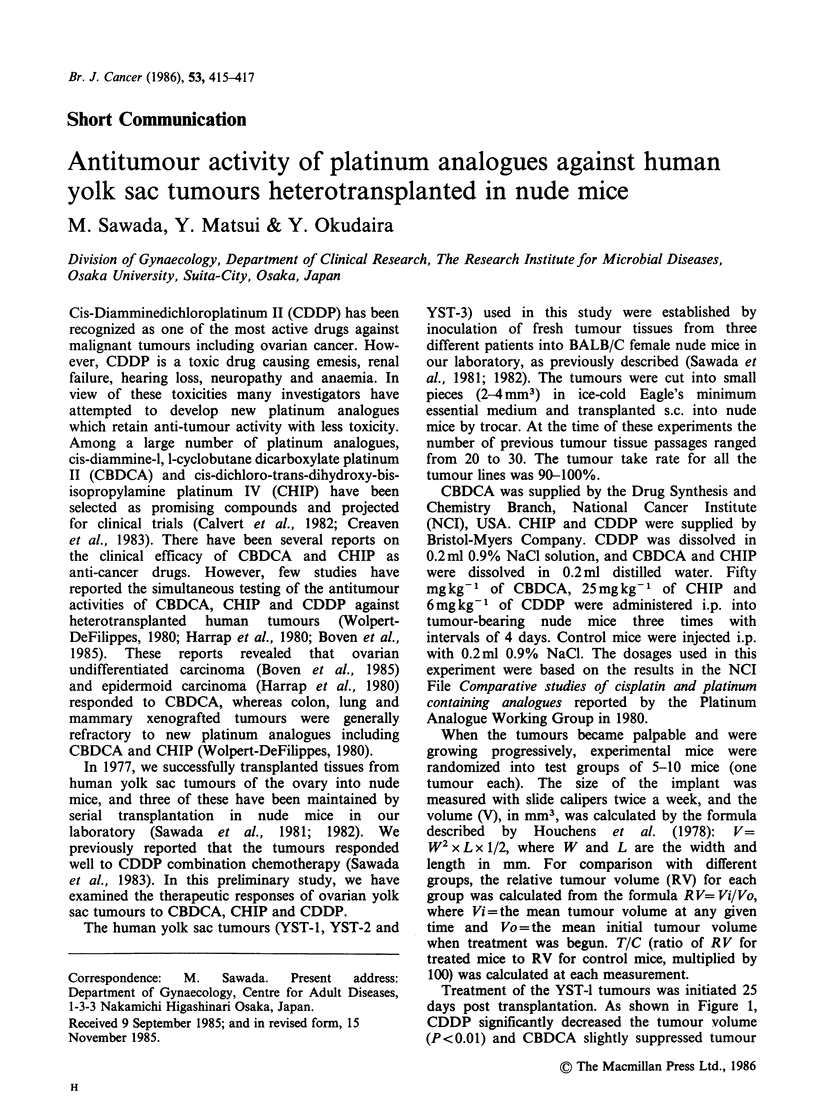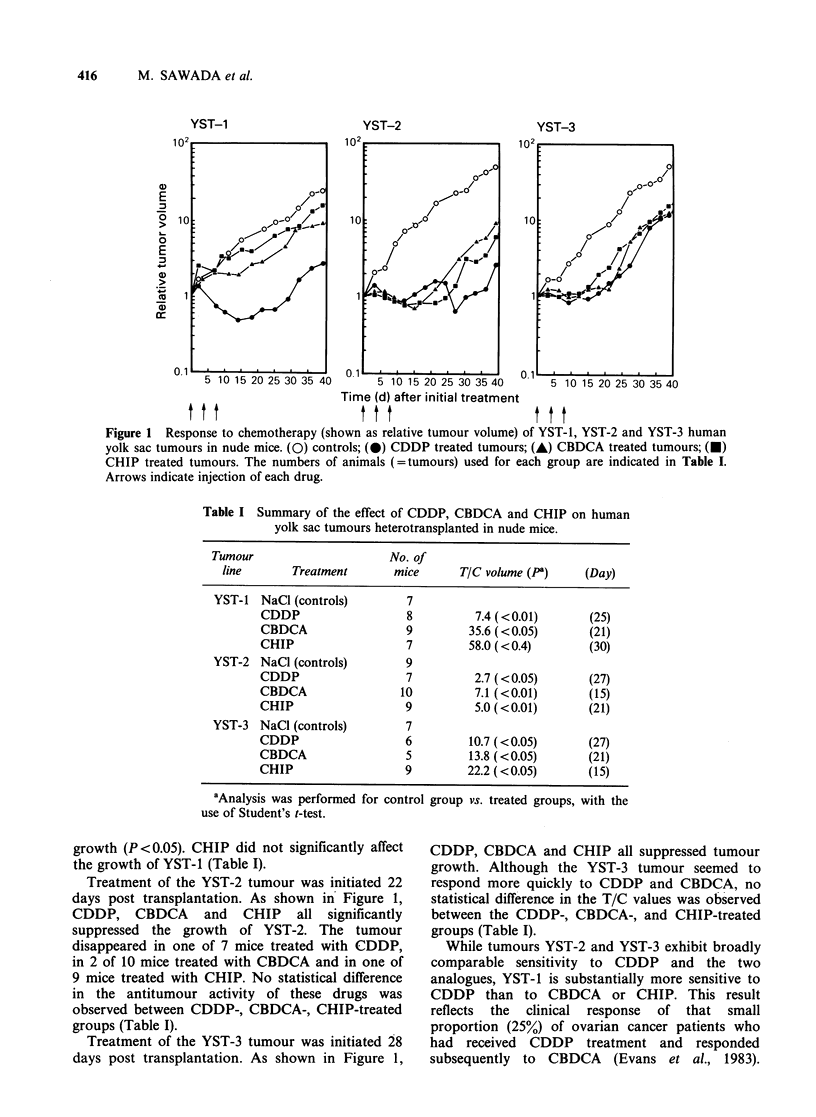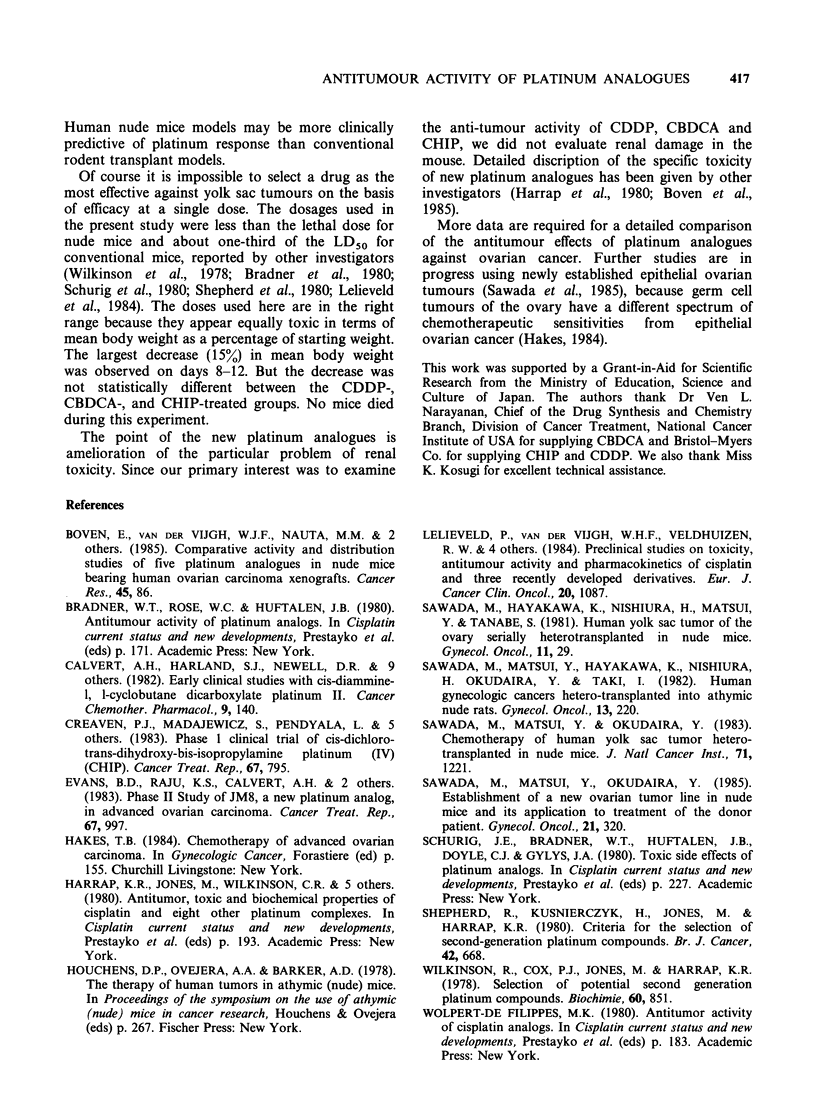# Antitumour activity of platinum analogues against human yolk sac tumours heterotransplanted in nude mice.

**DOI:** 10.1038/bjc.1986.68

**Published:** 1986-03

**Authors:** M. Sawada, Y. Matsui, Y. Okudaira


*Br. J. Cancer* (1986), **53**, 415–417

### Short Communication

### Antitumour activity of platinum analogues against human yolk sac tumours heterotransplanted in nude mice

M. Sawada, Y. Matsui & Y. Okudaira

*Division of Gynaecology, Department of Clinical Research, The Research Institute for Microbial Diseases, Osaka University, Suita-City, Osaka, Japan*

Cis-Diamminedichloroplatinum II (CDDP) has been recognized as one of the most active drugs against malignant tumours including ovarian cancer. However, CDDP is a toxic drug causing emesis, renal failure, hearing loss, neuropathy and anaemia. In view of these toxicities many investigators have attempted to develop new platinum analogues which retain anti-tumour activity with less toxicity. Among a large number of platinum analogues, cis-diammine-l, l-cyclobutane dicarboxylate platinum II (CBDCA) and cis-dichloro-trans-dihydroxy-bis-isopropylamine platinum IV (CHIP) have been selected as promising compounds and projected for clinical trials (Calvert *et al.*, 1982; Creaven *et al.*, 1983). There have been several reports on the clinical efficacy of CBDCA and CHIP as anti-cancer drugs. However, few studies have reported the simultaneous testing of the antitumour activities of CBDCA, CHIP and CDDP against heterotransplanted human tumours (Wolpert-DeFilippes, 1980; Harrap *et al.*, 1980; Boven *et al.*, 1985). These reports revealed that ovarian undifferentiated carcinoma (Boven *et al.*, 1985) and epidermoid carcinoma (Harrap *et al.*, 1980) responded to CBDCA, whereas colon, lung and mammary xenografted tumours were generally refractory to new platinum analogues including CBDCA and CHIP (Wolpert-DeFilippes, 1980).

In 1977, we successfully transplanted tissues from human yolk sac tumours of the ovary into nude mice, and three of these have been maintained by serial transplantation in nude mice in our laboratory (Sawada *et al.*, 1981; 1982). We previously reported that the tumours responded well to CDDP combination chemotherapy (Sawada *et al.*, 1983). In this preliminary study, we have examined the therapeutic responses of ovarian yolk sac tumours to CBDCA, CHIP and CDDP.

The human yolk sac tumours (YST-1, YST-2 and YST-3) used in this study were established by inoculation of fresh tumour tissues from three different patients into BALB/C female nude mice in our laboratory, as previously described (Sawada *et al.*, 1981; 1982). The tumours were cut into small pieces (2–4 $mm^3$) in ice-cold Eagle's minimum essential medium and transplanted s.c. into nude mice by trocar. At the time of these experiments the number of previous tumour tissue passages ranged from 20 to 30. The tumour take rate for all the tumour lines was 90–100%.

CBDCA was supplied by the Drug Synthesis and Chemistry Branch, National Cancer Institute (NCI), USA. CHIP and CDDP were supplied by Bristol-Myers Company. CDDP was dissolved in 0.2 ml 0.9% NaCl solution, and CBDCA and CHIP were dissolved in 0.2 ml distilled water. Fifty $mg\,kg^{-1}$ of CBDCA, 25 $mg\,kg^{-1}$ of CHIP and 6 $mg\,kg^{-1}$ of CDDP were administered i.p. into tumour-bearing nude mice three times with intervals of 4 days. Control mice were injected i.p. with 0.2 ml 0.9% NaCl. The dosages used in this experiment were based on the results in the NCI File *Comparative studies of cisplatin and platinum containing analogues* reported by the Platinum Analogue Working Group in 1980.

When the tumours became palpable and were growing progressively, experimental mice were randomized into test groups of 5–10 mice (one tumour each). The size of the implant was measured with slide calipers twice a week, and the volume (V), in $mm^3$, was calculated by the formula described by Houchens *et al.* (1978): $V = W^2 \times L \times 1/2$, where $W$ and $L$ are the width and length in mm. For comparison with different groups, the relative tumour volume (RV) for each group was calculated from the formula $RV = Vi/Vo$, where $Vi$ = the mean tumour volume at any given time and $Vo$ = the mean initial tumour volume when treatment was begun. $T/C$ (ratio of $RV$ for treated mice to RV for control mice, multiplied by 100) was calculated at each measurement.

Treatment of the YST-1 tumours was initiated 25 days post transplantation. As shown in Figure 1, CDDP significantly decreased the tumour volume ($P < 0.01$) and CBDCA slightly suppressed tumour

Correspondence: M. Sawada. Present address: Department of Gynaecology, Centre for Adult Diseases, 1-3-3 Nakamichi Higashinari Osaka, Japan.

Received 9 September 1985; and in revised form, 15 November 1985.

© The Macmillan Press Ltd., 1986

**Figure 1** Response to chemotherapy (shown as relative tumour volume) of YST-1, YST-2 and YST-3 human yolk sac tumours in nude mice. (○) controls; (●) CDDP treated tumours; (▲) CBDCA treated tumours; (■) CHIP treated tumours. The numbers of animals (=tumours) used for each group are indicated in **Table I**. Arrows indicate injection of each drug.

**Table I** Summary of the effect of CDDP, CBDCA and CHIP on human yolk sac tumours heterotransplanted in nude mice.

| *Tumour line* | *Treatment* | *No. of mice* | *T/C volume* ($P^a$) | *(Day)* |
|---|---|---|---|---|
| YST-1 | NaCl (controls) | 7 | | |
| | CDDP | 8 | 7.4 (<0.01) | (25) |
| | CBDCA | 9 | 35.6 (<0.05) | (21) |
| | CHIP | 7 | 58.0 (<0.4) | (30) |
| YST-2 | NaCl (controls) | 9 | | |
| | CDDP | 7 | 2.7 (<0.05) | (27) |
| | CBDCA | 10 | 7.1 (<0.01) | (15) |
| | CHIP | 9 | 5.0 (<0.01) | (21) |
| YST-3 | NaCl (controls) | 7 | | |
| | CDDP | 6 | 10.7 (<0.05) | (27) |
| | CBDCA | 5 | 13.8 (<0.05) | (21) |
| | CHIP | 9 | 22.2 (<0.05) | (15) |

[a]Analysis was performed for control group *vs.* treated groups, with the use of Student's *t*-test.

growth ($P<0.05$). CHIP did not significantly affect the growth of YST-1 (Table I).

Treatment of the YST-2 tumour was initiated 22 days post transplantation. As shown in Figure 1, CDDP, CBDCA and CHIP all significantly suppressed the growth of YST-2. The tumour disappeared in one of 7 mice treated with CDDP, in 2 of 10 mice treated with CBDCA and in one of 9 mice treated with CHIP. No statistical difference in the antitumour activity of these drugs was observed between CDDP-, CBDCA-, CHIP-treated groups (Table I).

Treatment of the YST-3 tumour was initiated 28 days post transplantation. As shown in Figure 1, CDDP, CBDCA and CHIP all suppressed tumour growth. Although the YST-3 tumour seemed to respond more quickly to CDDP and CBDCA, no statistical difference in the T/C values was observed between the CDDP-, CBDCA-, and CHIP-treated groups (Table I).

While tumours YST-2 and YST-3 exhibit broadly comparable sensitivity to CDDP and the two analogues, YST-1 is substantially more sensitive to CDDP than to CBDCA or CHIP. This result reflects the clinical response of that small proportion (25%) of ovarian cancer patients who had received CDDP treatment and responded subsequently to CBDCA (Evans *et al.*, 1983).

Human nude mice models may be more clinically predictive of platinum response than conventional rodent transplant models.

Of course it is impossible to select a drug as the most effective against yolk sac tumours on the basis of efficacy at a single dose. The dosages used in the present study were less than the lethal dose for nude mice and about one-third of the $LD_{50}$ for conventional mice, reported by other investigators (Wilkinson *et al.*, 1978; Bradner *et al.*, 1980; Schurig *et al.*, 1980; Shepherd *et al.*, 1980; Lelieveld *et al.*, 1984). The doses used here are in the right range because they appear equally toxic in terms of mean body weight as a percentage of starting weight. The largest decrease (15%) in mean body weight was observed on days 8–12. But the decrease was not statistically different between the CDDP-, CBDCA-, and CHIP-treated groups. No mice died during this experiment.

The point of the new platinum analogues is amelioration of the particular problem of renal toxicity. Since our primary interest was to examine the anti-tumour activity of CDDP, CBDCA and CHIP, we did not evaluate renal damage in the mouse. Detailed discription of the specific toxicity of new platinum analogues has been given by other investigators (Harrap *et al.*, 1980; Boven *et al.*, 1985).

More data are required for a detailed comparison of the antitumour effects of platinum analogues against ovarian cancer. Further studies are in progress using newly established epithelial ovarian tumours (Sawada *et al.*, 1985), because germ cell tumours of the ovary have a different spectrum of chemotherapeutic sensitivities from epithelial ovarian cancer (Hakes, 1984).

This work was supported by a Grant-in-Aid for Scientific Research from the Ministry of Education, Science and Culture of Japan. The authors thank Dr Ven L. Narayanan, Chief of the Drug Synthesis and Chemistry Branch, Division of Cancer Treatment, National Cancer Institute of USA for supplying CBDCA and Bristol–Myers Co. for supplying CHIP and CDDP. We also thank Miss K. Kosugi for excellent technical assistance.

**References**

BOVEN, E., VAN DER VIJGH, W.J.F., NAUTA, M.M. & 2 others. (1985). Comparative activity and distribution studies of five platinum analogues in nude mice bearing human ovarian carcinoma xenografts. *Cancer Res.*, **45**, 86.

BRADNER, W.T., ROSE, W.C. & HUFTALEN, J.B. (1980). Antitumour activity of platinum analogs. In *Cisplatin current status and new developments*, Prestayko *et al.* (eds) p. 171. Academic Press: New York.

CALVERT, A.H., HARLAND, S.J., NEWELL, D.R. & 9 others. (1982). Early clinical studies with cis-diammine-l, l-cyclobutane dicarboxylate platinum II. *Cancer Chemother. Pharmacol.*, **9**, 140.

CREAVEN, P.J., MADAJEWICZ, S., PENDYALA, L. & 5 others. (1983). Phase 1 clinical trial of cis-dichloro-trans-dihydroxy-bis-isopropylamine platinum (IV) (CHIP). *Cancer Treat. Rep.*, **67**, 795.

EVANS, B.D., RAJU, K.S., CALVERT, A.H. & 2 others. (1983). Phase II Study of JM8, a new platinum analog, in advanced ovarian carcinoma. *Cancer Treat. Rep.*, **67**, 997.

HAKES, T.B. (1984). Chemotherapy of advanced ovarian carcinoma. In *Gynecologic Cancer*, Forastiere (ed) p. 155. Churchill Livingstone: New York.

HARRAP, K.R., JONES, M., WILKINSON, C.R. & 5 others. (1980). Antitumor, toxic and biochemical properties of cisplatin and eight other platinum complexes. In *Cisplatin current status and new developments*, Prestayko *et al.* (eds) p. 193. Academic Press: New York.

HOUCHENS, D.P., OVEJERA, A.A. & BARKER, A.D. (1978). The therapy of human tumors in athymic (nude) mice. In *Proceedings of the symposium on the use of athymic (nude) mice in cancer research*, Houchens & Ovejera (eds) p. 267. Fischer Press: New York.

LELIEVELD, P., VAN DER VIJGH, W.H.F., VELDHUIZEN, R. W. & 4 others. (1984). Preclinical studies on toxicity, antitumour activity and pharmacokinetics of cisplatin and three recently developed derivatives. *Eur. J. Cancer Clin. Oncol.*, **20**, 1087.

SAWADA, M., HAYAKAWA, K., NISHIURA, H., MATSUI, Y. & TANABE, S. (1981). Human yolk sac tumor of the ovary serially heterotransplanted in nude mice. *Gynecol. Oncol.*, **11**, 29.

SAWADA, M., MATSUI, Y., HAYAKAWA, K., NISHIURA, H. OKUDAIRA, Y. & TAKI, I. (1982). Human gynecologic cancers hetero-transplanted into athymic nude rats. *Gynecol. Oncol.*, **13**, 220.

SAWADA, M., MATSUI, Y. & OKUDAIRA, Y. (1983). Chemotherapy of human yolk sac tumor hetero-transplanted in nude mice. *J. Natl Cancer Inst.*, **71**, 1221.

SAWADA, M., MATSUI, Y., OKUDAIRA, Y. (1985). Establishment of a new ovarian tumor line in nude mice and its application to treatment of the donor patient. *Gynecol. Oncol.*, **21**, 320.

SCHURIG, J.E., BRADNER, W.T., HUFTALEN, J.B., DOYLE, C.J. & GYLYS, J.A. (1980). Toxic side effects of platinum analogs. In *Cisplatin current status and new developments*, Prestayko *et al.* (eds) p. 227. Academic Press: New York.

SHEPHERD, R., KUSNIERCZYK, H., JONES, M. & HARRAP, K.R. (1980). Criteria for the selection of second-generation platinum compounds. *Br. J. Cancer*, **42**, 668.

WILKINSON, R., COX, P.J., JONES, M. & HARRAP, K.R. (1978). Selection of potential second generation platinum compounds. *Biochimie*, **60**, 851.

WOLPERT-DE FILIPPES, M.K. (1980). Antitumor activity of cisplatin analogs. In *Cisplatin current status and new developments*, Prestayko *et al.* (eds) p. 183. Academic Press: New York.